# Analysis of risk factors for acute kidney injury in children with severe wasp stings

**DOI:** 10.1007/s00467-023-06265-6

**Published:** 2024-01-10

**Authors:** Jing Lu, Liqun Dong, Lijuan Zhang, Yannan Guo, Hanmin Liu, Yang Liu

**Affiliations:** 1 Department of Pediatric Pulmonology and Immunology, West China Second University Hospital, Sichuan University, Chengdu, China; 2 Key Laboratory of Birth Defects and Related Diseases of Women and Children (Sichuan University), Ministry of Education, Chengdu, China; 3 NHC Key Laboratory of Chronobiology, (Sichuan University), Chengdu, China; 4 The Joint Laboratory for Lung Development and Related Diseases of West China Second University HospitalSichuan University and School of Life Sciences of Fudan University, West China Institute of Women and Children’s Health, West China Second University Hospital, Sichuan University, Chengdu, China; 5 Sichuan Birth Defects Clinical Research Center, West China Second University Hospital, Sichuan University, Chengdu, China

**Keywords:** Acute kidney injury (AKI), Risk factor, Children, Wasp stings, Blood purification, Multiple organ dysfunction syndrome (MODS)

## Abstract

**Background:**

Acute kidney injury (AKI) is common in children with sepsis, chronic kidney disease, poisoning or other conditions. Wasp stings are recognized as an important etiology. Several retrospective studies have investigated AKI after wasp stings in adults, but research on children remains limited.

**Methods:**

The study included 48 children with multiple organ dysfunction syndrome after wasp stings. Demographic data, clinical manifestations, laboratory findings, management and clinical outcomes were collected, and analyzed to identify early indicators or risk factors for AKI.

**Results:**

20 children (41.7%) developed AKI, and 28 (58.3%) did not. Serum creatine levels elevated mostly within 24 h from stings in children with AKI (16/20, 80%). Compared with non-AKI group, AKI group exhibited more cases with cola-colored urine, jaundice, and had higher sting numbers/body surface area (BSA) and higher revised sequential organ failure assessment scores (rSOFA) as well as higher levels of C-reactive protein (CRP), alanine aminotransferase (ALT), aspartate aminotransferase (AST), total bilirubin (TBIL), lactate dehydrogenase (LDH), troponin (cTnI), creatine kinase (CK), and longer prothrombin time (PT). Both univariable and multivariable logistic regression analysis identified cola-colored urine as a potential early risk factor for AKI.

**Conclusions:**

The AKI group exhibited higher sting numbers/BSA, higher levels of CRP, ALT, AST, TBIL, LDH, cTnI, and CK, as well as longer PT (p < 0.05). Our findings also suggest that cola-colored urine may serve as an early indicator or potential risk factor for AKI after wasp stings in children, which is very easy to identify for first aiders or pediatricians.

**Graphical abstract:**

**Figure:**
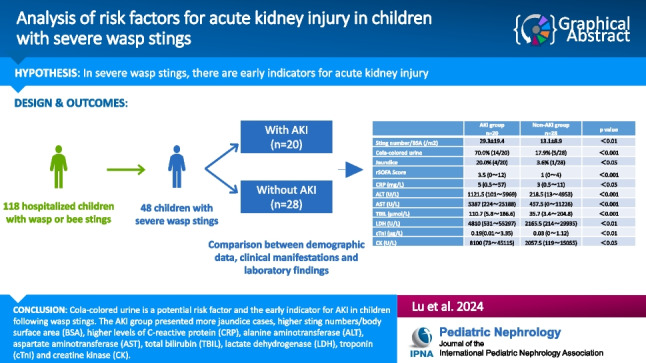
A higher resolution version of the Graphical abstract is available as Supplementary information

**Supplementary Information:**

The online version contains supplementary material available at 10.1007/s00467-023-06265-6.

## Introduction

Acute kidney injury (AKI) is common in children in nephrology departments and intensive care units (ICU), often resulting from infection, cardiovascular diseases, hypovolemia or other conditions. Wasp stings are also recognized as an important cause of AKI. Studies have shown that wasp stings are a growing public health problem worldwide with the global climate change, particularly in countries in Asia and Latin America, ever since the first report by Sitprijafrs & Boonpucknavigin, 1972 [1]. Zhang et al. reported a series of wasp sting cases in adults, revealing a mortality rate of 9.3%, and a rate of 10.7% progressing to chronic kidney disease (CKD) [2]. Dhanapriya et al. reported 11 cases with wasp sting-induced AKI in adults from India [3]. Xie et al. contributed the largest amount of data with wasp stings and concluded that the occurrence of AKI in wasp stings was 21.0%, based on a series of 1091 cases [4].

The mechanisms of wasp sting-induced AKI include direct venom toxicity to the kidney, renal tubular damage, obstruction by hemoglobulin and myoglobin or secondary inflammation induced by wasp-stings. In clinical practice, nephrologists are concerned about the risk factors for AKI induced by wasp stings, as timely treatment can make the prognosis completely different. However, the few studies addressing this issue are concerning adults. In a retrospective study of 508 adult patients with wasp stings, Tang et al. found several independent risk factors for AKI: the number of stings, aspartate aminotransferase (AST) > 147 U/L, lactate dehydrogenase (LDH) > 477 U/L, time from stings to admission > 12 h, and activated partial thromboplastin time (APTT) > 49 s [5]. In addition, Yuan suggested that elevated leukocytes, high myoglobin and high urinary monocyte chemotactic protein-1 (MCP-1) were independent risk factors for AKI following wasp stings [6]. For pediatricians, we are eager to obtain the relevant data focused on children. But so far, information about the risk factors of AKI in children with wasp stings is still lacking.

Outcomes vary according to the severity, ranging from complete recovery, to chronic kidney disease, to death. Vikrant et al. reported 10 deaths among 35 patients (10/35, 28.6%) due to acute respiratory distress syndrome (ARDS), massive bleeding, metabolic causes and stroke [7]. Zhang et al. reported 7 deaths among 75 patients (mortality rate 9.3%), 8 patients (10.7%) progressing to CKD, and 60 patients achieving complete recovery [2]. The situation in children still needs to be elucidated.

This study retrospectively analyzed the clinical data of 48 children who experienced severe wasp stings and developed multiple organ dysfunction syndrome (MODS). The aim was to identify risk factors for AKI, providing early predictors of AKI for pediatricians and nephrologists. We hypothesized that children with wasp stings might exhibit unique characteristics. As MODS itself was related to AKI and might be considered as a confounding factor, we just included the cases with MODS in conducting a stratified analysis. Thus, we focused solely on the children with MODS, aiming to obtain more accurate risk factors through this management strategy.

## Methods

### Patients

**Fig. 1:**
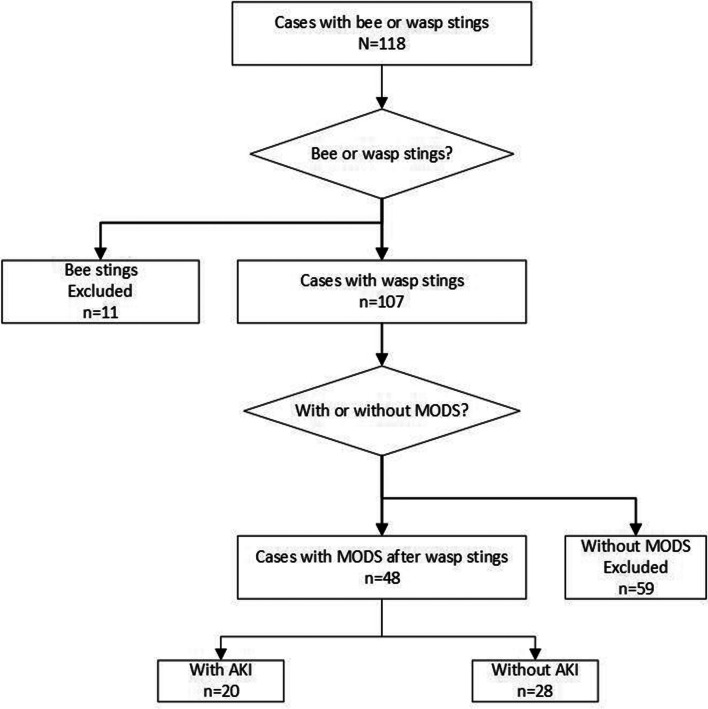
Case screening flowchart

This retrospective cohort study initially collected data of wasp or bee sting cases in the Pediatric nephrology department of West China Second Hospital of Sichuan University from January 2014 to January 2022. Yet, the cases involving bee stings were excluded because bee venom is much less toxic than wasp venom, thus we focused on the cases with wasp stings. Eventually only the children with severe wasp stings (defined as cases with MODS) were included. The study finally included 48 cases, categorized into two groups: the AKI group and the non-AKI group (Fig. 1).

This research was in accordance with the Medical Ethics Committee of West China Second Hospital of Sichuan University and complied with the 1964 Helsinki Declaration and its subsequent amendments.

### Definition of AKI in children

The diagnosis of AKI complied with Kidney Disease Improving Global Outcomes (KDIGO) guidelines (2012). KDIGO guidelines define AKI as a state with abnormal structure or function of the kidney, characterized by a sudden decline in kidney function. Specifically, AKI can be identified by an increase in serum creatinine of ≥ 0.3 mg/dl (> 26.5 μmol/L) within 48 h, an increase in serum creatinine levels ≥ 1.5 times the baseline within 7 days, or a urine output of < 0.5 ml/(kg.h) for a duration of 6 h. In our department, not all children had indwelling urethral catheters, thus it was hard for us to obtain real-time urine output. As such, serum creatinine levels ≥ 1.5 times baseline was chosen as the criterion for AKI. For a child with known baseline creatinine values within the last three months, we considered the patient’s lowest creatinine value as the baseline. However, for patients with unknown baseline values, we considered the lowest creatinine value during hospitalization or normal value from children with the same body weight as the baseline.

### Clinical manifestations

Demographic data, including age, gender, body weight, and blood pressure (both systolic blood pressure (SBP) and diastolic blood pressure (DBP)) were collected. Clinical manifestations such as cola-colored urine, vomiting, fatigue, lethargy, jaundice, headache, dizziness, fever, dyspnea, edema, irritability, wheal, coma, convulsion, anorexia, and abdominal pain were recorded. Hypertension was assessed according to the Guideline for Screening and Management of High Blood Pressure in Children and Adolescents [8]. Hypotension in SBP was defined as follows: 1 ~ 12 months < 70 mmHg, 1 ~ 10 years < 70 mmHg + 2 × age in years, 10 ~ 14 years < 90 mmHg [9]. Hypotension in DBP was defined as follows: 1 ~ 12 months < 40 mmHg, 1 ~ 10 years < 50 mmHg, 10 ~ 14 years < 60 mmHg. The sting numbers/body surface area (BSA) were calculated instead of recording the absolute numbers of stings, as we believed that sting number/BSA might be a more appropriate indicator to estimate the severity of wasp stings in children.

Laboratory findings, such as peripheral total white blood cell count (WBC), neutrophil count (N), hemoglobin (Hb), platelet count (Plt), C-reactive protein (CRP, serum), alanine aminotransferase (ALT, serum), aspartate aminotransferase (AST, serum), total bilirubin (TBIL, serum), conjugated bilirubin (DBIL, serum), unconjugated bilirubin (IBIL, serum), troponin (cTnI, serum), creatine kinase (CK, serum), myoglobin (Mb, serum), lactate dehydrogenase (LDH, serum), prothrombin time (PT, plasma), activated partial thromboplastin time (APTT, plasma) and blood glucose (Glu, serum) were also collected. Blood urea nitrogen (BUN, serum) and blood creatinine (Cr, serum) were not included as potential risk factors for AKI, as they were used for the definition of AKI.

### Sequential organ failure assessment scoring (SOFA)

SOFA scoring involved the detection and evaluation of various systems, including the respiratory function, nervous system, cardiovascular system, coagulation function, liver and kidney function. Each component was assigned a score ranging from 0 to 4 points, resulting in a total score of 24 points. A higher score indicated a more severe condition. However, considering that the kidney score might affect the SOFA results, we excluded it from the revised SOFA score (rSOFA).

### Management

For children with MODS, appropriate management is very important. In addition to supportive treatments such as removing the stingers, taking JiDesheng anti-venom tablets (a widely used Chinese herb in China), anti-allergic treatment, rehydration, maintaining electrolyte balance and nutritional support, blood purification treatments were deemed to be the most important therapy.

Each vascular access for blood purification was established by inserting Gambro single-needle double lumen catheter (6F, 8F or 11F) into the femoral vein or subclavian vein. There were four modes of blood purification treatments, including ⑴ hemoperfusion: performed using the Fresenius blood purification machine (5008S, Germany) and hemoperfusion column (YTS-100 or YTS-160, China). This method facilitates the removal of large molecular toxins by adsorption; ⑵ therapeutic plasma exchange (TPE): carried out using either the Prismaflex blood purification machine with its plasma separator (TPE1000 or TPE2000, Gambro, Germany) or the Multifiltrate blood purification machine with its plasma separator (P1 or P2, produced by Fresenius, Germany). Each TPE treatment involved removing plasma volume approximately 5% of the body weight and replacing it with an equal volume of fresh frozen plasma (FFP). TPE eliminates the toxins present in the blood plasma; ⑶sustained low efficiency dialysis (SLED): employed for children with an elevated level of serum nitrogen or creatine, by using the Fresenius hemodialysis machine (5008S, Germany). Although SLED has a lower clearing efficiency than continuous kidney replacement therapy (CKRT), it is a cost-effective option; ⑷ CKRT: utilized for children with AKI, volume overload, or acute pulmonary edema. Prismaflex or Multifiltrate blood purification machines were employed to manage these critical conditions. We recorded the modes and sessions in both groups, and compared them and the clinical outcomes between the groups. Besides, we also recorded the durations of hospitalization.

### Statistics

All data were processed using SPSS 23.0 statistical software (IBM Corporation, Armonk, NY, USA). The data were presented in the form of numbers (percentages), means ± standard deviation, or median (minimum, maximum) according to their distributions. Measurement data were analyzed using the t-test or Wilcoxon test, while categorical data were analyzed using Pearson χ^2^ test or Fisher’s exact test. Potential risk factors were analyzed by univariable and multivariable logistic regression. The association between potential risk factors and AKI was quantified using odds ratios (OR) and 95% confidence intervals (CI). P values < 0.05 were considered statistically significant.

## Results

### Basic information

From January 2014 to January 2022, a total of 118 cases of hospitalized children with wasp or bee stings in the Pediatric Nephrology department of West China Second Hospital of Sichuan University were enrolled. Among these cases, 48 children (48/118, 40.7%) experienced severe wasp stings (Fig. 1). The median age of the 48 children was 5.3 years (IQR: 2.8 ~ 10.3 years), median body weight was 18.8 kg (IQR: 15 ~ 29.8 kg) (Table 1).

**Table 1:** Clinical data comparison of 48 severe wasp sting cases with and without AKI

	AKI group *n* = 20	Non-AKI group *n* = 28	*p* value
Demographic data			
Age[(yr), M(Q_L_,Q_U_)]	7.6 ± 4.1	5.4 ± 3.5	> 0.05
Gender (male: female)	1.9: 1	1: 1	> 0.05
Weight (kg)	19.6 (11.5 ~ 59)	18 (8.5 ~ 52)	> 0.05
Sting number/BSA (/m^2^)	29.3 ± 19.4	13.1 ± 8.9	< 0.01
Blood pressure (mmHg)			
Systolic blood pressure (SBP)			
Hypotension	0	1 (3.5%)	
Normal	9 (45.0%)	14 (50.0%)	> 0.05
90% ≤ SBP < 95%	0	5 (17.9%)	
SBP ≥ 95%	11 (55.0%)	8 (28.6%)	
Diastolic blood pressure (DBP)			
Hypotension	1 (5.0%)	1 (3.6%)	
Normal	6 (30.0%)	18 (64.3%)	< 0.05
90% ≤ DBP < 95%	3 (15.0%)	5 (17.9%)	
DBP ≥ 95%	10 (50.0%)	4 (14.3%)	
Clinical manifestation			
Cola-colored urine	70.0% (14/20)	17.9% (5/28)	< 0.001
Vomiting	50.0% (10/20)	28.6% (8/28)	> 0.05
Fatigue	30.0% (6/20)	21.4% (6/28)	> 0.05
Lethargy	25.0% (5/20)	7.1% (2/28)	> 0.05
Jaundice	20.0% (4/20)	3.6% (1/28)	< 0.05
Headache	15.0% (3/20)	14.3% (4/28)	> 0.05
Dizziness	15.0% (3/20)	14.3% (4/28)	> 0.05
Fever	15.0% (3/20)	7.1% (2/28)	> 0.05
Dyspnea	15.0% (3/20)	7.1% (2/28)	> 0.05
Edema	10.0% (2/20)	10.7% (3/28)	> 0.05
Irritability	5.0% (1/20)	14.3% (4/28)	> 0.05
Wheal	5.0% (1/20)	7.1% (2/28)	> 0.05
Coma	5.0% (1/20)	3.6% (1/28)	> 0.05
Convulsion	5.0% (1/20)	0	> 0.05
Anorexia	0	3.6% (1/28)	> 0.05
Abdominal pain	0	10.7% (3/28)	> 0.05
rSOFA	3.5 (0 ~ 12)	1 (0 ~ 4)	< 0.001

*AKI*, acute kidney injury; *BSA*, body surface area; *SBP*, systolic blood pressure; *DBP*, diastolic blood pressure; *rSOFA*, revised SOFA score

Among the severe wasp sting cases, there were 27 males (56.2%) and 21 females (43.8%). The majority of the children experienced wasp stings in Autumn (August to November) (46/48, 95.8%). Blood pressure measurements revealed that SBP varied from 79 to 140 mmHg (mean 110.6 mmHg) and DBP from 45 to 97 mmHg (mean 65.3 mmHg). There was no significant difference between the two groups in terms of age, gender, weight, and SBP. However, a statistically significant difference was found in DBP, where the AKI group had relatively higher DBP (Table 1).

### Acute kidney injury

According to the 2012 KDIGO Guidelines for AKI, 20 children (41.7%) with severe wasp sting injuries were diagnosed with AKI. Among them, 9 children (45.0%) were classified as stage 1; 5 children (25.0%) stage 2; and 6 cases (30.0%) stage 3. The other 28 children (58.3%) did not develop AKI. Among the children with AKI, 80% exhibited elevated serum creatine levels within 24 h. The median time of serum creatine elevation in children after wasp stings was 17.5 h (5 ~ 75 h), and the median time for peak serum creatine value was 28.5 h (5, 137 h). Five cases in the AKI group and five cases in the non-AKI group reported prehospital oliguria, but oliguria or anuria was observed in only 7 cases from the AKI group during the initial 24 h of hospitalization. Urinary glucose was found to be positive in 5 cases with AKI (5/20, 25%) and 3 cases without AKI (3/28, 10.7%). However, in all the 8 cases, the urinary glucose disappeared within one week (1 ~ 7 d).

### Clinical manifestations

The 48 patients exhibited various clinical manifestations. All of the cases suffered pain in the sting areas (48/48, 100%), and nearly one-third had cola-colored urine (19/48, 39.6%) and vomiting (18/48, 37.5%). The specific clinical manifestations of both groups are listed in Table 1. Of note, most of the 19 patients with cola-colored urine (12/19, 63.2%) had no hematuria upon urinalysis, i.e. urine red blood cells (RBC) < 3/HP (high power field, × 400). Five children had slightly elevated RBC in urine (urine RBC 3 ~ 10/HP, × 400), and two children had significantly elevated RBC with urine RBC 3 +  ~ 4 + /HP (3 + : urine red blood cells seen up to 3/4 per HP, × 400; 4 + : urine RBC seen in each HP, × 400). CK was also analyzed among the 19 cases with cola-colored urine, and we found an interesting phenomenon: the average level of CK in the 2 cases with significant hematuria (26,557.5 U/L) was much higher than that of the 12 cases (7597.5 U/L) without hematuria, suggesting that the higher the CK level was, the more severe the hematuria. The numbers of wasp stings were recorded, ranging from 1 to 75 stings. Two children with 1 sting were a 4-month-old boy and 7-month-old girl. The sting numbers/BSA varied from 1.36/m^2^ to 74.4/m^2^, and the ratios were significantly higher in AKI group (P < 0.01) (Table 1).

Revised SOFA scores were also recorded within the first 24 h after the wasp stings in the 48 children. In total, rSOFA scores in the AKI group (median 3.5) were higher than those in the non-AKI group (median 1).

There were significant differences in incidences of cola-colored urine and jaundice, in the sting numbers/BSA and rSOFA scores between the two groups. The AKI group appeared to have higher incidences of cola-colored urine and jaundice, higher sting numbers/BSA and higher rSOFA scores (Table 1).

### Changes in laboratory indicators

Laboratory findings included WBC (× 10^9^/L), N (× 10^9^/L), Hb (g/L), Plt (× 10^9^/L), CRP (mg/L), ALT (U/L), AST (U/L), TBIL (μmol/L), IBIL (μmol/L), DBIL (μmol/L), LDH (U/L), cTnI (μg/L), CK (U/L), Mb (μg/L), PT (s), APTT (s) and Glu (mmol/L). The results showed statistically significant differences between the AKI and non-AKI groups in CRP, ALT, AST, TBIL, LDH, UN, Scr, cTnI, CK, and PT. Compared with the non-AKI group, the AKI group exhibited significantly elevated ALT, AST, TBIL, DBIL, LDH, and CK levels and moderately elevated CRP, cTnI IBIL, and PT levels (Table 2).

**Table 2:** Laboratory findings comparison of severe wasp sting children with or without AKI

	AKI group *n* = 20	Non-AKI group *n* = 28	*p* value
WBC (× 10^9^/L)	23.8 ± 10.0	21.3 ± 8.1	> 0.05
N (× 10^9^/L)	17.6 (6 ~ 45.1)	17.8 (5.9 ~ 36)	> 0.05
Hb (g/L)	115.2 ± 27.5	120.7 ± 10.3	> 0.05
Plt (× 10^9^/L)	283.5 (58.0 ~ 739.0)	296.0 (203.0 ~ 620.0)	> 0.05
CRP (mg/L)	5 (0.5 ~ 57)	3 (0.5 ~ 11)	< 0.05
ALT (U/L)	1121.5 (101 ~ 5969)	218.5 (13 ~ 4953)	< 0.001
AST (U/L)	5387 (224 ~ 25,188)	457.5 (0 ~ 11,226)	< 0.001
TBIL (μmol/L)	110.7 (5.8 ~ 186.6)	35.7 (3.4 ~ 204.8)	< 0.001
IBIL (μmol/L)	59.4 ± 33.9	34.6 ± 21.2	< 0.05
DBIL (μmol/L)	46.6 (0 ~ 100)	0 (0 ~ 100)	< 0.001
LDH (U/L)	4810 (531 ~ 55,297)	2165.5 (214 ~ 29,935)	< 0.01
cTnI (μg/L)	0.19 (0.01 ~ 3.35)	0.03 (0 ~ 1.12)	< 0.01
CK (U/L)	8100 (73 ~ 45,115)	2057.5 (119 ~ 15,055)	< 0.05
Mb (µg/L)	2100 (15 ~ 31,000)	2057.5 (119 ~ 15,055)	> 0.05
PT (s)	14.7 (11.4 ~ 23.9)	13.1 (10.9 ~ 15.0)	< 0.05
APTT (s)	56.2 (25.4 ~ 300.0)	50.2 (22.0 ~ 89.8)	> 0.05
Glu (mmol/L)	6.8 (3.6 ~ 31.5)	7.8 (5.0 ~ 10.7)	> 0.05

*WBC*, the blood count of white blood cells; *N*, neutrophils; *Hb*, hemoglobin; *Plt*, platelet; *CRP*, C-reactive protein; *ALT*, alanine aminotransferase; *AST*, aspartate aminotransferase; *TBIL*, total bilirubin; *IBIL*, indirect bilirubin; *DBIL*, direct bilirubin; *LDH*, lactate dehydrogenase; *cTnI*, troponin; *CK*, creatine kinase; *Mb*, myoglobin; *PT*, prothrombin time; *APTT*, activated partial thromboplastin time; *Glu*, blood glucose

### Logistic regression

In the multiple rounds of multivariable logistic regression analysis, due to the limited sample size, two to three confounders were included each round. All confounders included age, gender, weight, clinical manifestations and laboratory indicators. In each round of multivariable logistic regression analysis, cola-colored urine was always found to be significantly different between the two groups, thus was considered to be a potential risk factor for AKI with MODS (p < 0.05). However, jaundice and sting numbers/BSA could not be considered as potential risk factors for AKI (p > 0.05) (Table 3).

**Table 3:** Univariable and multivariable logistic regression analysis of AKI

Potential risk factors	Univariable logistic regression			Multivariable logistic regression		
	OR	95%CI	*p*	OR	95%CI	*p*
Cola-colored urine	10.733	[2.754, 41.824]	0.001	8.904	[2.198, 36.075]	0.002
Jaundice	6.750	[0.693, 65.787]	0.100	4.096	[0.309, 54.334]	0.285
Sting number/BSA	2.860	[0.388, 21.091]	0.303	3.265	[0.290, 36.829]	0.338

*AKI*, acute kidney injury; *OR*, odds ratio; *CI*, confidence interval; p, *p* value

### Management and prognosis

Blood purification treatment: The modes and sessions of blood purification treatment depended on the clinical manifestations and the consents from the guardians of the patients. In AKI group (n = 20), a total of 106 sessions were performed in 18 patients (most cases underwent more than one session): 3 hemoperfusions (3/106, 2.8%), 42 TPE (42/106, 39.6%), 13 SLED (13/106, 12.3%), and 48 CKRT (48/106, 45.3%). Thus, among these sessions, mostly TPE and CKRT were performed in the AKI group. Of the 8 cases who underwent a single mode of blood purification, 5 cases receiving only TPE recovered within 3 ~ 5 days; 2 cases receiving only SLED and having increased creatinine levels did not recover in one-month follow-up period; and 1 case receiving only CKRT recovered within 8 d. Of the 10 cases who underwent more than one mode of blood purification (each mode 1 ~ 17 sessions), 1 patient died; 6 patients recovered in 7 ~ 11 d; and the other 3 patients’ creatinine did not recover in one-month follow-up period. Of the 2 cases who did not receive any blood purification, one patient died quickly after admission and the other refused to receive any therapy and was lost to follow-up. In the non-AKI group (n = 28), 46 sessions of blood purification treatment were performed: 4 hemoperfusions (4/46, 8.7%), 39 TPE (39/46, 84.8%), 0 SLED and 3 CKRT (3/46, 6.5%). Of the non-AKI cases, 2 underwent more than one mode of blood purification (each mode 1 ~ 3 sessions); 15 underwent only one mode of blood purification (each mode 2 ~ 4 sessions); and 11 did not receive any blood purification. All of the cases in non-AKI group recovered and were discharged within 2 weeks. Regarding hospitalization durations, the average length of hospital stay in the AKI group was 13.5 d, compared to 6.2 d in the non-AKI group (P < 0.05). Of the two patients who died with AKI (2/20, 10%), one was a five-year-old boy who died from cardiac arrest, and the other was a four-year-old boy who died from acute pulmonary edema. No death was found in the non-AKI group.

## Discussion

It has been reported that patients suffering from wasp stings often develop AKI. However, AKI induced by wasp stings has its own characteristics compared to AKI in other conditions, such as sepsis-associated acute kidney injury [10], obstructive nephropathy, underlying chronic renal diseases, etc. In this study, the pediatric cases did not have any underlying diseases and the wasp stings were the only pathogenic factors.

Although there are almost 5,000 species of wasp known all over the world, the components of venom are similar. Wasp venom is a transparent complicated mixture stored in a venom sac, and is 20 times more toxic than bee venom. Wasp venom’s chemical composition includes amines (melittin, histamine, dopamine), peptides (kinin, mast cell degranulation polypeptide), and high molecular weight proteins (neurotoxic protein, phospholipase A_2_, hyaluronidase) [11]. The most important substances leading to organ lesions are melittin, phospholipase A_2_, hyaluronidase, and antigen 5 [12]. Melittin, which has been recently found to play an active role in cancer and infection [13–15], is an antimicrobial peptide that strongly damages cell membranes, leading to leakage of cell contents, and eventually cell death [16, 17]. It can also cause central nervous system damage through the blood–brain barrier due to its small molecular weight [18]. Phospholipase A_2_ mainly cooperates with melittin to cause acute hemolysis. Both phospholipase A_2_ and histamine can cause allergic reactions [19]. Undoubtedly, these toxins can stimulate the body to produce inflammatory reactions. Li et al. found that the levels of IL-2, IL-6, IL-10, TNF-α and IL-17 were elevated in wasp sting patients; in particular, IL-6, IL-10 and IL-17 levels were significantly increased in the AKI group compared with the non-AKI group [20]. Both the toxins in the venom and secondary inflammatory factors can lead to damage to vital organs like the kidney.

AKI is a common phenomenon in patients following wasp stings and attracts attention from physicians and researchers. Vikrant and Parashar reported a series of 32 adults with wasp stings, and concluded that AKI was a serious complication with a high mortality rate (50%), and that severe cases might progress to MODS, affecting liver, lung, kidney, and coagulation systems [7, 21]. Although concerned about AKI in wasp stings, few researchers have investigated the risk factors associated with AKI. In a retrospective study conducted by Liu et al., which included 1,131 adults with wasp stings, it was suggested that tea-colored urine, number of stings, LDH, and TBIL were risk factors for developing a severe case of wasp stings [22]. After observation of 11 farmers with multiple wasp stings, Dhanapriya et al. concluded that AKI was most commonly associated with rhabdomyolysis [3]. On the analysis of 112 adult patients with hospital admissions resulting from wasp stings, Yuan et al. suggested that high leukocytes, high myoglobin, and high urinary MCP-1 upon admission, were risk factors for AKI [6]. This study is the first study focusing on children with AKI induced by severe wasp stings. We found that cola-colored urine, oliguria, jaundice. and higher rSOFA scores, as well as a higher level of CRP, ALT, AST, TBIL, LDH, cTnI, CK and PT might be candidate risk factors for AKI in children.

In order to exclude confounding factors, this study only included children with severe wasp stings who developed MODS. Children without MODS were excluded. The children with MODS were divided into the AKI group and the non-AKI group, ensuring conditions of both groups were well-balanced. Although this management resulted in exclusion of some cases with wasp stings, it might be better for accurate identification of potential risk factors for AKI in wasp sting patients.

Previously known pathogenesis of AKI in wasp sting patients included hypotension, which was thought to play an important role in prerenal acute renal insufficiency. However, recent findings suggested that hypotension was not as common as previously thought [23–25]. This study suggested that hypertension was more common in children affected by wasp stings and hypotension or hypoperfusion caused by the inflammatory response in wasp sting patients did not play a major role in the mechanism of AKI.

Cola-colored urine is a prominent clinical manifestation after wasp stings. In a series of adult cases, Wang et al. suggested that macroscopic hematuria could be considered as a surrogate marker of deteriorating clinical outcomes in wasp sting patients [26]. In this study, cola-colored urine was identified as a potential risk factor for AKI in children following wasp stings. Cola-colored urine, occurring as an early symptom, can also be considered an early predictor of AKI after multiple wasp stings. Here we described cola-colored urine instead of macroscopic hematuria. As in this study, 19 children exhibited cola-colored urine, but the majority (12 cases) showed no hematuria by urinalysis (urine RBC < 3/HP) with 5 children having slightly elevated RBC in urine (urine RBC 3 ~ 10/HP) and only 2 children having significantly elevated RBC in urine (urine RBC 3 +  ~ 4 + /HP). It at least indicated that glomerular injury did not play a major role in the occurrence of AKI, which is consistent with the previously reported kidney injury mechanism [27–29]. For rural village hospitals or health centers, this early indicator does not require a laboratory test and is very easy for rural doctors to judge, to estimate the high risk of kidney injury, and to make timely and correct decisions on referral [30].

Jaundice, as another symptom after wasp stings, appeared to be a risk factor according to our χ^2^ test, but logistic regression did not show a significant difference. Different from the study by Yuan et al. [6], no significant differences in the levels of WBC, N, Hb, and PLT were observed between AKI and non-AKI groups, possibly due to the inclusion of only MODS cases in our study.

In our data, the fact that one sting induced MODS in 2 children (4-month-old boy and 7-month-old girl) attracted our attention, and we realized the absolute sting numbers might not be appropriate in assessing the sting severity in children. Different from the variables in adults, we introduced the concept of the sting number/BSA, due to the fact that children are constantly growing individuals and there are significant differences among children. From the results in this study, the median ratio of the sting number/BSA in AKI group (mean 29.3/m^2^) was more than 2 times of that in non-AKI group (mean 13.1/m^2^).

Pathology of kidney biopsy in AKI patients after wasp stings has been investigated by researchers. Based on literature reports, wasp sting patients showed mostly mild tubulointerstitial nephritis [31, 32], while others showed pigment nephropathy [33], mesangial proliferative glomerulonephritis [34], acute tubular necrosis [35–37], minimal-change disease [38], and glomerulonephritis. In some rare conditions, acute renal cortical necrosis occurred.

Though this is the first study to analyze the risk factors of AKI in children after wasp stings, it has some limitations. First, the limited number of cases might lead to ignorance of some other risk factors, thus more studies and cases are needed to address this issue. Second, we did not include the data concerning markers of tubular damage and urine myoglobin. Third, kidney biopsy was not performed in these children and would otherwise undoubtedly enhance our knowledge of wasp sting-induced AKI in children.

## Conclusion

In conclusion, this study retrospectively explored the risk factors of AKI in 48 pediatric cases with MODS after wasp stings. The findings suggest that cola-colored urine is a potential risk factor and an early indicator for AKI in children following wasp stings. Additionally, the AKI group presented more jaundice cases, higher sting numbers/BSA, higher levels of CRP, ALT, AST, TBIL, LDH, cTnI, and CK. Therefore, the findings are helpful for pediatricians to identify cases with a higher risk of AKI and enable prompt management of this serious condition.

### Supplementary information

Below is the link to the electronic supplementary material.

Graphical abstract (PPTX 48 KB)

## Data Availability

All data included in this study are available upon request by contact with the corresponding author.

## Abbreviations

*ALT*: Alanine aminotransferase
*APTT*: Activated partial thromboplastin time
*ARDS*: Acute respiratory distress syndrome
*AST*: Aspartate aminotransferase
*BSA*: Body surface area
*BUN*: Blood urea nitrogen
*CK*: Creatine kinase
*CKD*: Chronic kidney disease
*CRP*: C-reactive protein
*CKRT*: Continuous kidney replacement therapy
*cTnI*: Troponin
*DBIL*: Conjugated bilirubin
*IBIL*: Unconjugated bilirubin
*LDH*: Lactate dehydrogenase
*MCP-1*: Monocyte chemotactic protein-1
*MODS*: Multiple organ dysfunction syndrome
*PT*: Prothrombin time
*rSOFA*: Revised SOFA
*SLED*: Sustained low efficiency dialysis
*SOFA*: Sequential organ failure assessment scoring
*TBIL*: Total bilirubin
*TPE*: Therapeutic plasma exchange
