# Supplementary material for: Analysis of risk factors for acute kidney injury in children with severe wasp stings
Source: Pediatr Nephrol. 2024 Jan 10;39(6):1927–35. doi: 10.1007/s00467-023-06265-6 (PMC11026182; doi:10.1007/s00467-023-06265-6)
Supplement: Supplementary file 1 — Graphical abstract (PPTX 48 KB) [file 467_2023_6265_MOESM1_ESM.pptx]

## Slide 1
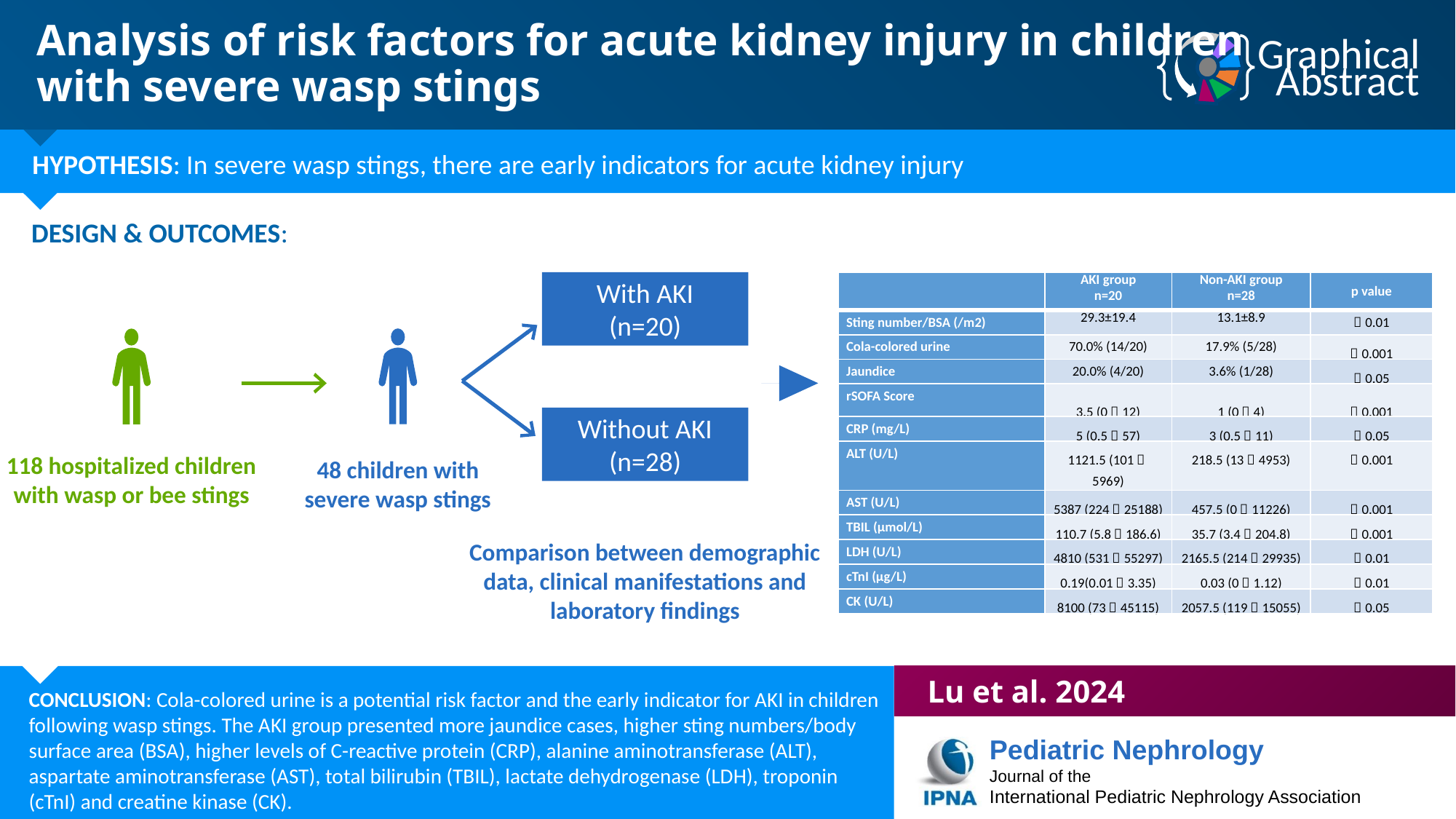

Analysis of risk factors for acute kidney injury in children
with severe wasp stings
HYPOTHESIS: In severe wasp stings, there are early indicators for acute kidney injury
DESIGN & OUTCOMES:
With AKI
(n=20)
| | AKI group n=20 | Non-AKI group n=28 | p value |
| --- | --- | --- | --- |
| Sting number/BSA (/m2) | 29.3±19.4 | 13.1±8.9 | ＜0.01 |
| Cola-colored urine | 70.0% (14/20) | 17.9% (5/28) | ＜0.001 |
| Jaundice | 20.0% (4/20) | 3.6% (1/28) | ＜0.05 |
| rSOFA Score | 3.5 (0～12) | 1 (0～4) | ＜0.001 |
| CRP (mg/L) | 5 (0.5～57) | 3 (0.5～11) | ＜0.05 |
| ALT (U/L) | 1121.5 (101～5969) | 218.5 (13～4953) | ＜0.001 |
| AST (U/L) | 5387 (224～25188) | 457.5 (0～11226) | ＜0.001 |
| TBIL (μmol/L) | 110.7 (5.8～186.6) | 35.7 (3.4～204.8) | ＜0.001 |
| LDH (U/L) | 4810 (531～55297) | 2165.5 (214～29935) | ＜0.01 |
| cTnI (μg/L) | 0.19(0.01～3.35) | 0.03 (0～1.12) | ＜0.01 |
| CK (U/L) | 8100 (73～45115) | 2057.5 (119～15055) | ＜0.05 |
Without AKI (n=28)
118 hospitalized children with wasp or bee stings
48 children with severe wasp stings
Comparison between demographic data, clinical manifestations and laboratory findings
Lu et al. 2024
CONCLUSION: Cola-colored urine is a potential risk factor and the early indicator for AKI in children following wasp stings. The AKI group presented more jaundice cases, higher sting numbers/body surface area (BSA), higher levels of C-reactive protein (CRP), alanine aminotransferase (ALT), aspartate aminotransferase (AST), total bilirubin (TBIL), lactate dehydrogenase (LDH), troponin (cTnI) and creatine kinase (CK).
